# The intraoral permeability measurement as a screening for artifact formation by orthodontic products in MRI

**DOI:** 10.1007/s00056-021-00360-9

**Published:** 2021-11-04

**Authors:** Felix H. Blankenstein, Ulrike Kielburg, Ludwig Melerowitz, Daniel Stelmaszczyk

**Affiliations:** 1 Department of Prosthodontics, Geriatric Dentistry and Craniomandibular Disorders, Charité—Universitätsmedizin Berlin, Corporate Member of Freie Universität Berlin, Humboldt-Universität zu Berlin and Berlin Institute of Health, Aßmannshauser Str. 4–6, 14197 Berlin, Germany; 2 Branch office Stendal, Clinic for Radio oncology, Johanniter Ltd., Wendstraße 31, 39576 Stendal, Germany

**Keywords:** Magnetic resonance imaging, Brackets, Validity, Reliability, Artifact-screening, Magnetresonanztomografie, Brackets, Validität, Reliabilität, Artefakt-Screening

## Abstract

**Aim:**

Metal dental products lack precautionary statements regarding MR compatibility due to an exemption in the labelling obligation. Hence, it is difficult for radiologists to decide whether to remove fixed metal objects in patients prior to MRI. A solution could be the direct determination of the magnetic permeability (µ_r_) as a decisive material-related predictor of artifact formation and other interactions. Thus, the applicability of an industrially used measurement device as a screening instrument and the relevance of the manufacturer’s application restrictions in vitro and in vivo were tested.

**Methods:**

Precision and trueness were tested using self-made test objects with different dimensions and different permeability. To clarify whether the measurement results are affected by the remanence (B_R_) induced in the objects, 28 brackets of different materials were exposed to a weak and a strong external magnetic field and the magnetic flux density before and after these exposures was compared. The clinical test was performed on a volunteer with an orthodontic appliance experimentally composed of brackets with different levels of magnetic permeability (µ_r_). Validity and intra- and interrater reliability were calculated using two rater groups consisting of four dentists and four medical-technical radiology assistants (MTRA), respectively.

**Results:**

With coefficients of variation below 0.14%, precision was excellent regardless of object surface and size. Trueness was high on objects with µ_r_ ≤ 1.002, and decreased with increasing µ_r_, for which size-dependent correction factors were calculated. Intra- and interrater reliability and validity were excellent and independent of professional intraoral manipulation experience.

**Conclusions:**

The permeability measurement allows for a valid and reliable determination of the magnetizability of intraoral metal objects. When used as a screening tool to detect nonartifact-causing objects, no correction factor needs to be calculated. For the first time, it offers radiologists a decision support for the selective removal of only the highly permeable components of the multiband apparatus.

## Abstract (de)

**Ziel:**

Bei zahnmedizinischen Produkten aus Metall fehlen aufgrund einer Ausnahmeregelung in der Kennzeichnungspflicht Vorsichtshinweise zur MR(Magnetresonanz)-Kompatibilität. Daher ist es für Radiologen schwierig zu entscheiden, ob festsitzende Metallobjekte bei Patienten vor der MRT (Magnetresonanztomografie) entfernt werden sollen. Eine mögliche Lösung könnte die direkte Bestimmung der magnetischen Permeabilität (µ_r_) als ein entscheidender materialbedingter Prädiktor für Artefaktbildung und andere Wechselwirkungen sein. Daher prüften wir die Eignung eines industriell genutzten Messgerätes als Screeninginstrument und untersuchten die Relevanz der vom Hersteller angegebenen Einsatzbeschränkungen in vitro und in vivo.

**Methoden:**

Zur Prüfung von Präzision und Richtigkeit dienten selbstgefertigte Prüfkörper mit unterschiedlichen Dimensionen und unterschiedlicher Permeabilität. Zur Klärung, ob eine in den Objekten induzierte Remanenz (B_R_) die Messergebnisse beeinträchtigt, wurden 28 Brackets aus unterschiedlichen Materialien einem schwachen und einem starken Fremdmagnetfeld ausgesetzt und die magnetische Flussdichte vor und nach diesen Expositionen verglichen. Die klinische Prüfung fand an einem Probanden mit einer experimentell aus unterschiedlich permeablen Brackets zusammengesetzten orthodontischen Apparatur statt. Für die Berechnung von Validität sowie Intra- und Interrater-Reliabilität wurden 2 Untersuchergruppen aus je 4 Zahnärzten und medizinisch-technischen Radiologieassistenten (MTRA) gebildet.

**Ergebnisse:**

Mit Variationskoeffizienten unter 0,14 % war die Präzision unabhängig von Objektoberfläche und Größe exzellent. Die Richtigkeit war auf Objekten mit µr ≤ 1,002 hoch. Sie verringerte sich mit steigendem µ_r_, wofür größenabhängige Korrekturfaktoren berechnet wurden. Die Intra- und Interrater-Reliabilität sowie Validität waren exzellent und unabhängig von beruflicher Erfahrung mit intraoraler Manipulation.

**Schlussfolgerungen:**

Die Permeabilitätsmessung ermöglicht eine valide und reliable Bestimmung der Magnetisierbarkeit intraoraler Metallobjekte. Wenn sie als Screeninginstrument zur Erkennung nicht Artefakt verursachender Objekte eingesetzt wird, bedarf es keines Korrekturfaktors. Sie bietet dem Radiologen auch erstmals eine Entscheidungshilfe für die selektive Entfernung einzelner hochpermeabler Komponenten der Multibandapparatur.

## Introduction

Patients with medical implants can only be legally examined in an MRI if these products can be unmistakably identified and are labeled by the manufacturer as “MR safe” or “MR conditional” including the conditions to be met [1]. According to the EU Medical Devices Regulation 2017/745, information on the behavior of a medical device in a magnetic field must be provided in the instructions for use [24]. However, chapter II, article 18 (3) exempts some products used in dentistry and oral surgery from this obligation, including fixed orthodontic appliances. Radiologists thus lack the simplest source of information for assessing the risks emanated by these metal objects in MRI. At least, there are more reliable data on the potential heating and force effects: brackets heat up by less than 1 °C, while wires were found to increase by a maximum of 3 °C [11, 20, 22, 26]. Forces acting on correctly anchored brackets and wires are also harmless, which is about a factor of 1000 below the force required for debonding [13].

The data on the formation of susceptibility artifacts is much less reliable. This is because a systematic error runs through the relevant literature: often only individual products are examined as representative of the entire product range. Therefore the authors generalize that steel products should be generally removed for artifact-free diagnostics [2, 5, 8, 17, 18, 21, 25]. This coincides with the everyday experience at the MRI, where brackets made of magnetizable steel grades can erase the entire visceral cranium or even render the entire imaging system unusable, depending on the artifact susceptibility of the MRI sequence. However, this ignores the wide range of steels that can be used for orthodontic products, including the stable nonmagnetic steel grades that can remain in situ during MR imaging. A solution to this lack of information could be a direct intraoral test of magnetizability expressed as the relative magnetic permeability µ_r_. This dimensionless parameter is defined as the ratio µ/µ_0_ (permeability of a specific medium/vacuum permeability) and describes the magnetizability of a material in response to an external magnetic field.

The relative magnetic permeability (µ_**r**_) is the crucial material-related predictor for the occurrence of susceptibility artifacts [3]. From the commercially available measuring instruments, we selected an easy-to-use device, the “Ferromaster” (Stefan Mayer Instruments, Dinslaken, Germany). Its measuring range extends to values between 1.001 and 1.999 with a resolution of 0.001. In the probe head, a permanent magnet generates a constant field of 35 kA/m. Two field probes measure the field inhomogeneity generated during placement, from which µ_r_ of the object is calculated. The measuring deviation is given with f = (μ_r_ −1) × 5% [23]. The device was originally designed for quality control of nonmagnetizable stainless steels, e.g. in the vicinity of electron microscopes and MRI devices.

In a previous study with orthodontic products in a chambered phantom, we used the Ferromaster and found a correlation between µ_r_ and the artifact extension for each a typical spin echo and gradient echo sequence [4]. However, such studies are still not available for the many other sequences and their modifications. In the present study, we now wanted to investigate the extent to which direct permeability measurement can be used to make a valid and reliable decision about leaving or removing small medical metal objects before MRI.

**Fig. 1:**
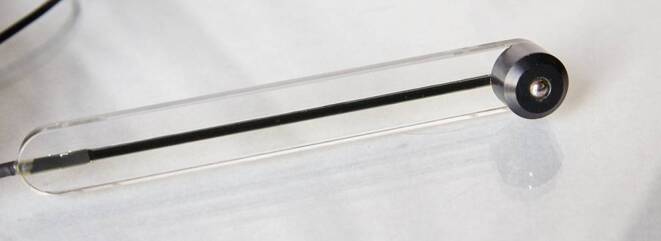
The modified probe head Der modifizierte Tastkopf

In order to improve handling and hygiene during intraoral use, the manufacturer modified the probe head of the device purchased for this study so that it differed from the series production (Fig. 1).

However, the manufacturer specifies three application restrictions to ensure the measuring accuracy:

- The objects should have a flat surface.
- The objects should have a minimum size of d = 20 mm and h = 5 mm.
- Objects that have already been exposed to a magnetic field should be demagnetized before permeability measurement due to the induced remanence (B_R_) [23].

Yet, these limitations affect the whole range of metallic brackets and wires: they are all significantly smaller than the minimum size (further described as “undersized”), their surfaces facing the oral cavity are not flat, and it is usually unknown whether they have already been exposed to an external magnetic field.

The present study was intended to firstly clarify the relevance of these limitations for clinical use on orthodontic and similar metal objects by determining the precision and trueness of the measurement method on regular and undersized objects under laboratory conditions. Subsequently, the intra- and interrater reliability and the validity of the measurement was determined under clinical conditions. The influence of additionally ligated arch wires on the one hand and the dental professional experience of the raters on the other hand was investigated.

Finally, it was the aim to clarify whether the method is suitable for a screening instrument for the magnetizability of intraoral metal objects.

### Materials and methods

#### In vitro precision

Three objects of different sizes were used for this purpose, including one with an uneven surface (Table 1). After initial zeroing of the device, µ_r_ of each of these objects were measured 20 times. We used the coefficient of variation (CV), which is calculated as the quotient of the standard deviation and mean value and is expressed as a percentage. Values below 10% are considered as high precision, whereas a CV greater than 30% indicates an unacceptably scattering of the method [19].

**Table 1:** Determination of the precision of the Ferromaster measurement Bestimmung der Präzision der Ferromaster-Messung

Objects	20 measurements x̅ of µ_r_ ± SD	CV (in %)
Cylindrical calibration body of the Ferromaster with µ_r_ = 1.36 h = 9 mm/d = 49 mm—plane surface	1.360 ± 0.00136	0.10
Cylindrical undersized test specimen h = 3.6 mm/d = 7.8 mm—plane surface	1.274 ± 0.00179	0.14
Steel bracket Empower self-ligating 11 (American Orthodontics) h = 2.2 mm/l = 3.4 mm—rugged surface	1.280 ± 0.00146	0.11

*SD* standard deviation, *CV* coefficient of variation, *h* height, *d* depth

#### In vitro trueness (accuracy of the mean)

**Fig. 2:**
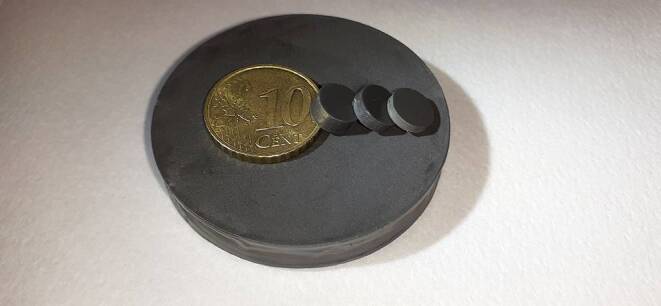
One of the 26 groups of specimens for determination of in vitro trueness. They each consisted of one sufficiently large reference body and three significantly undersized test specimens. The coin illustrates the size ratios. The physical data are shown in Table 3 Eine der 26-Proben-Gruppen zur Bestimmung der In-vitro-Richtigkeit. Sie bestanden jeweils aus einem ausreichend großen Referenzkörper und 3 deutlich unterdimensionierten Probekörpern. Die Münze veranschaulicht die Größenverhältnisse. Die physikalischen Daten sind in Tab. 3 aufgeführt

By mixing the cold-curing PMMA plastic Technovit 4004 (Kulzer Ltd., Hanau, Germany) with varying amounts of a carbonyl iron powder, a total of 26 masses were produced. Depending on the iron content, their µ_r_ value was between 1.00 and 1.96. Four cylinders were cast from each of these masses: One reference body each with diameter (d) = 45 mm and height (h) = 8 mm, which exceeds the required minimum size, and three undersized test specimens each with d = 7.8 mm and the different heights of 1.6, 2.6 and 3.6 mm (Fig. 2). On a total of 78 test specimens we then measured µ_r_, compared it with the values of the 26 associated reference bodies and evaluated the differences mathematically. The set values of the reference bodies were compared with the actual values of the test specimens. A correction factor was determined based on the gradient of the compensation line resulting from the distribution of the points.

#### Influence of remanence (B_R_)

Two specimens each of 14 commercially available metal brackets made of different alloys (Table 2), which according to the manufacturer’s specifications had not yet been exposed to a magnetic field, served as test specimens. To check whether a remanent magnetization was already present, the existing flux density (measurement A) was determined initially. The device Teslameter FM 220 (Projekt Elektronik Mess- und Regelungstechnik GmbH, Berlin, Germany) was used for this purpose. Its measuring deviation is stated as ±0.5% + 2 digits at 25 °C. As a second step, the initial value of µ_r_ was determined (measurement D). This step also served as the first exposition of the brackets in an external magnetic field, the approx. 0.04 T strong field of the Ferromaster probe. The subsequent measurement of the flux density (measurement B) now showed whether a remanent field was induced in the brackets and at what level.

**Table 2:** Induction of remanence in different brackets and its influence on the relative permeability: the used products and alloys, their flux density (A, B, C) and magnetic permeability (D, E) before and after exposure in external fields^b^ Induktion einer Remanenz in verschiedenen Brackets und ihr Einfluss auf die relative Permeabilität: Die verwendeten Produkte und Legierungen, ihre Flussdichte (A, B, C) und magnetische Permeabilität (D, E) vor und nach Exposition in externen Feldern^b^

No.	Name	Manufacturer reference number	Material steel grade: AISI/DIN structure	A initial flux density (mT)^a^	B remanent flux density after Ferromaster exposure (mT)^a^	C remanent flux density after 1.9 T exposure (mT)^a^	D permeability µ_r_ initial value^a^	E permeability µ_r_ after exposure at 1.9 T^a^
1	Equilibrium® Ti	Dentaurum (Ispringen/Germany) 722-501-11	Titanium	0.01 ± 0.02	0.01 ± 0.02	0.01 ± 0.02	1.000 ± 0.001	1.000 ± 0.001
				0.01 ± 0.02	0.01 ± 0.02	0.01 ± 0.02	1.000 ± 0.001	1.000 ± 0.001
2	Rematitan® buccal tube	Dentaurum 723-020-00	Titanium	0.01 ± 0.02	0.01 ± 0.02	0.01 ± 0.02	1.000 ± 0.001	1.000 ± 0.001
				0.01 ± 0.02	0.01 ± 0.02	0.01 ± 0.02	1.000 ± 0.001	1.000 ± 0.001
3	Topic	Dentaurum 790-312-00	Cobalt chrome molybdenum	0.01 ± 0.02	0.01 ± 0.02	0.01 ± 0.02	1.000 ± 0.001	1.000 ± 0.001
				0.01 ± 0.02	0.01 ± 0.02	0.01 ± 0.02	1.000 ± 0.001	1.000 ± 0.001
4	Clarity™ SL 3er	3M™ Unitek (Landsberg a. Lech/Germany) 3007-406	Al_2_O_3_-ceramic inside: 316/1.4401—stable austenite	0.01 ± 0.02	0.01 ± 0.02	0.01 ± 0.02	1.000 ± 0.001	1.000 ± 0.001
				0.01 ± 0.02	0.01 ± 0.02	0.01 ± 0.02	1.000 ± 0.001	1.000 ± 0.001
5	Discovery® delight	Dentaurum 790-451-00	Steel 316L/1.4404 metastable austenite	0.01 ± 0.02	0.01 ± 0.02	0.01 ± 0.02	1.000 ± 0.001	1.000 ± 0.001
				0.01 ± 0.02	0.01 ± 0.02	0.01 ± 0.02	1.000 ± 0.001	1.000 ± 0.001
6	Discovery® smart	Dentaurum 722-902-11	Steel 316L/1.4404 metastable austenite	0.01 ± 0.02	0.01 ± 0.02	0.01 ± 0.02	1.000 ± 0.001	1.000 ± 0.001
				0.01 ± 0.02	0.01 ± 0.02	0.01 ± 0.02	1.000 ± 0.001	1.000 ± 0.001
7	Equilibrium®-2	Dentaurum 722-301-21	Steel 316L/1.4404 metastable austenite	0.01 ± 0.02	0.01 ± 0.02	0.01 ± 0.02	1.000 ± 0.001	1.000 ± 0.001
				0.01 ± 0.02	0.01 ± 0.02	0.01 ± 0.02	1.000 ± 0.001	1.000 ± 0.001
8	Equilibrium® mini	Dentaurum 718-442-13	Steel 304/1.4301 metastable austenite steel 316L/1.4404 metastable austenite	0.01 ± 0.02	0.01 ± 0.02	0.01 ± 0.02	1.000 ± 0.001	1.000 ± 0.001
				0.00 ± 0.02	0.01 ± 0.02	0.01 ± 0.02	1.000 ± 0.001	1.000 ± 0.001
9	Victory™ Tube 6er	3M™ Unitek 3067-9613	Steel 316L/1.4404 metastable austenite	0.06 ± 0.02	0.03 ± 0.02	0.02 ± 0.02	1.344 ± 0.017	1.338 ± 0.017
				0.01 ± 0.02	0.04 ± 0.02	0.03 ± 0.02	1.325 ± 0.016	1.332 ± 0.017
10	Bracket Saturn Roth	Dental Vertrieb 2000 (Alsbach-Hähnlein/Germany) 300-S05-31	Steel 304/1.4301 metastable austenite	0.01 ± 0.02	0.48 ± 0.02	0.20 ± 0.02	1.472 ± 0.024	1.472 ± 0.024
				0.01 ± 0.02	0.38 ± 0.02	0.24 ± 0.02	1.483 ± 0.024	1.476 ± 0.024
11	Metallbrackets Roth	Dental Vertrieb 2000 300-05-35 H	Steel 304/1.4301 metastable austenite	0.05 ± 0.02	0.37 ± 0.02	0.83 ± 0.02	1.393 ± 0.020	1.392 ± 0.020
				0.02 ± 0.02	0.45 ± 0.02	0.18 ± 0.02	1.384 ± 0.019	1.378 ± 0.018
12	Empower ® Self Ligating	American Orthodontics (Weil/Germany) 475-1117	Steel 630/1.4542 martensite	0.03 ± 0.02	0.04 ± 0.02	0.02 ± 0.02	1.259 ± 0.013	1.263 ± 0.013
				0.01 ± 0.02	0.07 ± 0.02	0.10 ± 0.02	1.249 ± 0.013	1.249 ± 0.013
13	Master Series mini™	American Orthodontics 390-1014	Steel 630/1.4542 martensite	0.03 ± 0.02	0.24 ± 0.02	0.28 ± 0.02	1.217 ± 0.011	1.221 ± 0.011
				0.01 ± 0.02	0.26 ± 0.02	0.23 ± 0.02	1.168 ± 0.008	1.174 ± 0.009
14	Midi Low Friction	ODS GmbH (Kisdorf/Germany) 1002-641000	Steel 630/1.4542 martensite	0.04 ± 0.02	0.62 ± 0.02	0.57 ± 0.02	1.421 ± 0.021	1.424 ± 0.021
				0.04 ± 0.02	0.47 ± 0.02	0.50 ± 0.02	1.422 ± 0.021	1.431 ± 0.022

^a^Measured values with variability display which is always less than or equal to the measurement deviation specified by the manufacturer

^b^Product numbers (rows) 5–8: metastable austenitic products, made by metal injection moulding (MIM). Product numbers (rows) 9–11: metastable austenitic products, made by cold-forming

*AISI* American Iron and Steel Institute, *DIN* Deutsche Industrienorm

The second exposure was performed in the main field of a 1.5 T MRI Aera (Siemens AG, Healthcare Sector, Erlangen, Germany). Its point of maximum field strength of 1.9 T is located in the tunnel behind the upper gantry opening. Brackets were positioned for about six seconds in this “worst case” zone. Then the final determination of B_R_ (measurement C) and µ_r_ (measurement E) was performed.

#### Validity and inter-/intrarater reliability

Two deep-drawn bite splints made of polymethyl methacrylate (PMMA) with vestibularly attached brackets in the 1st, 2nd and 3rd quadrant were made for an adult test person with naturally healthy teeth. The products selected for this purpose were not equivalent to a clinically applicable device, but they were assembled exclusively on the basis of their different µ_r_ values between 1.001 and 1.444 (measured under laboratory conditions, uncorrected). In the 1st quadrant a 16-round steel arch made of the metastable austenitic steel 1.4310 was ligated. In the 2nd quadrant a 17/25 titanium–molybdenum (TMA) arch was used, the 3rd quadrant remained without arch. With 21 brackets and two wires a total of 23 measuring points were obtained. The R‑package ICC.sample.size was used to calculate the required number of examiners. We assumed an ICC of 0.5 and for the null hypothesis the value 0 with *n* = 23 and α = 0.05 (two-sided). This resulted in a power of over 90% for four examiners. Two groups of examiners were formed, who differed in their professional experience with manipulations in the oral cavity: four dentists employed at a university dental clinic and four medical-technical radiology assistants (MTRA) from a university radiological institute, each with several years of professional experience.

Initially, a 15 min briefing was given on the aim and procedure of the study and on the handling of the measuring device. During the measurements, the test person sat on a simple chair without headrest. No additional instruments were used. At the beginning and at the end of the measurement in each quadrant the device was zeroed. As soon as the examiner had placed the probe head stably on the respective object, he announced the display value. At intervals of 3 weeks, two repeat measurements were carried out under identical conditions. The study was single blinded: Only the study director, who was not involved in recording the measurement results, knew the magnetic properties of the objects used.

Statistical evaluation was performed using SPSS 23.0 (IBM, Armonk, NY, USA). The Cronbach’s alpha (α) method was used to quantify the reliability, with values > 0.9 indicating excellent agreement [6]. After evaluation of a normal distribution of all measurement series, the single Pearson’s correlation coefficient *r* was applied to compare the intraoral measurement values with the laboratory values (validity). Values between 0.8 and 1 represent a strong to perfect positive correlation [9]. The reliability of the measurement series of the individual examiners was tested by comparing the mean values with the corresponding laboratory values. The 5% level was defined as the significance threshold.

## Results

### In vitro precision

The very low values of the coefficients of variation showed a very high precision both on the calibration body and on the significantly smaller and additionally nonplanar test specimen. Taking into account the device measurement deviation, there was no difference between the results for the objects (Table 1).

### In vitro trueness (accuracy of mean)

**Fig. 3:**
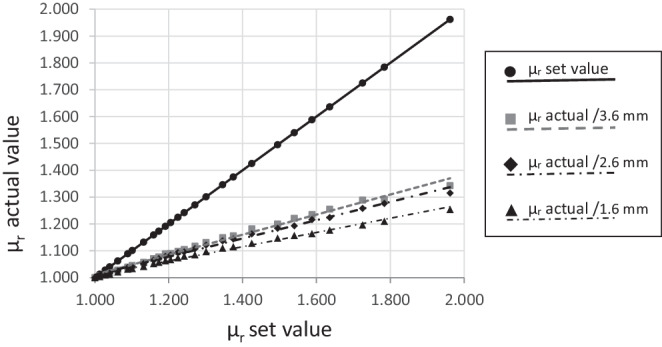
Comparison of set values and actual values of the relative permeability measured on the 26 reference bodies and the respective three undersized test specimens Vergleich von Soll- und Istwerten der relativen Permeabilität, gemessen an den 26 Referenzkörpern und den jeweils 3 unterdimensionierten Prüfkörpern

Table 3 shows the increasing µ_r_ values of the 26 reference bodies from 1.000 to 1.962 (set values) and the three corresponding test specimens of different heights (here called actual values). Even with the cylinders made from mass two, there was a small difference between the set values and the smaller actual values. This difference increased both with thinner test pieces and with increasing permeability number of the 26 masses. Fig. 3 compares the three actual value curves of the test specimens with the set value curve of the reference bodies. A compensation line y = m × x + b was generated, which necessarily runs through the y‑axis segment b = 1. Its gradient shows the percentage of the actual values from the corresponding set value. The reciprocal value of this gradient thus corresponds to the respective correction factor K:

- at h = 3.6 mm: 39.4%; K = 2.54
- at h = 2.6 mm: 35.9%; K = 2.78
- at h = 1.6 mm: 28.3%; K = 3.54

**Table 3:** Relative permeability of the correctly dimensioned reference bodies and three corresponding undersized test specimens each made of 26 different PMMA–iron mixtures Relative Permeabilität der korrekt dimensionierten Referenzkörper und dreier zugehöriger unterdimensionierter Probekörper aus jeweils 26 verschiedenen PMMA-Eisen-Mischungen

PMMA–iron mixture No.	*µ*_*r*_ of the reference body “set values”	*µ*_*r*_ of the three undersized test specimens “actual value” with		
		h = 3.6 mm	h = 2.6 mm	h = 1.6 mm
1	1.000	1.000	1.000	1.000
2	1.013	1.006	1.005	1.004
3	1.028	1.014	1.012	1.010
4	1.040	1.018	1.017	1.013
5	1.062	1.028	1.025	1.021
6	1.089	1.039	1.038	1.032
7	1.101	1.045	1.040	1.034
8	1.132	1.057	1.052	1.042
9	1.159	1.071	1.065	1.052
10	1.173	1.077	1.071	1.057
11	1.191	1.089	1.079	1.063
12	1.205	1.090	1.084	1.067
13	1.225	1.098	1.093	1.074
14	1.242	1.105	1.096	1.080
15	1.271	1.117	1.108	1.085
16	1.301	1.131	1.121	1.097
17	1.346	1.149	1.137	1.110
18	1.375	1.156	1.144	1.115
19	1.425	1.182	1.163	1.128
20	1.495	1.200	1.184	1.147
21	1.540	1.221	1.193	1.157
22	1.588	1.235	1.216	1.164
23	1.636	1.255	1.224	1.177
24	1.725	1.289	1.258	1.196
25	1.784	1.292	1.276	1.210
26	1.962	1.343	1.315	1.254

*PMMA* polymethyl methacrylate, *h* height

**Fig. 4:**
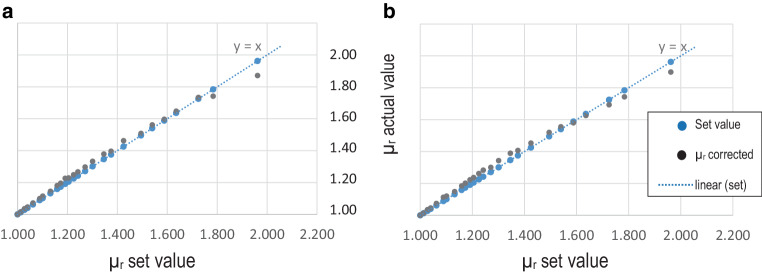
Corrected actual values with µ_r_ corr = 1 + (µ_r_ actual −1) × K. **a** Test specimens height 3.6 mm / K = 2.54, **b** Test specimens height 1.6 mm / K = 3.54 Korrigierte Ist-Werte mit µ_r_ corr = 1 + (µ_r_ actual −1) × K. **a** Prüfkörperhöhe 3,6 mm / K = 2,54, **b** Prüfkörperhöhe 1,6 mm / K = 3,54

The respective correction factor (K) for the selected test specimen height must be entered into the resulting formula for the calculation of the correct permeability number: µ_r corrected_ = 1 + (µ_r actual_ − 1) × K. This showed that, taking into account the measuring deviation of the Ferromaster, the observed differences each have a constant factor. As shown in Fig. 4, the corrected actual values of the two exemplary selected test specimen heights of 1.6 and 3.6 mm approximately lie on the nominal straight line, indicating that the correction formulae are accurate. The plot of the set and actual straight lines shows that no correction is necessary for a measured permeability number µ_r actual_ ≤ 1.002.

### Influence of remanence

With regard to magnetizability, the 14 different bracket types in Table 2 can be divided into two groups: The brackets listed under numbers 1–8 consist of various materials: titanium, CoCrMo alloy, ceramic or metastable austenitic steels. They can all be identified as paramagnetic with µ_r_ = 1.000 by means of the measurement D. They do not amplify an externally applied magnetic field and are considered nonmagnetic by definition. Accordingly, no residual magnetic field could be detected in these brackets after exposure to weak or strong external fields (measurement B and C). All measurements up to 0.01 mT lay within the measuring deviation. The bracket numbers 9–14 are made from metastable austenitic or martensitic steels. Measurement D with µ_r_ >> 1 proved them to be ferromagnetic. A weak remanent field of max. 0.6 mT was induced by contact with the probe (measurement B), which did not further increase after the second exposure at 1.9 T (measurement C). µ_r_ did not change after MRI exposure in all 14 bracket types (measurement E) compared to the initial value (measurement D). Any differences that occurred lay within the measuring deviation. Thus, the induction of a remanent field did not affect the results of the permeability measurement.

### Reliability and validity

First, the interrater reliability within the two groups was calculated across all 23 measuring points (Table 4). The group of the dentists showed excellent agreement in their measurements indicated by a Cronbach’s alpha (α) of 0.992. The correlations between the individual dentists lay between 0.975 and 0.960. There were no significant differences between the four group members. In the group of the MTRAs, a slightly lower but also excellent agreement was achieved with α = 0.979. With correlations of 0.974 and 0.865 they showed greater interindividual differences than the dentists.

**Table 4:** Agreement of measured values between the examiners and between repeated examinations with joint and separate calculation of the quadrants Übereinstimmung der Messwerte zwischen den Untersuchern und zwischen wiederholten Untersuchungen bei summarischer und bei separater Berechnung der Quadranten

Cronbach’s α	Summarized calculation	Separate calculation of the 1st, 2nd and 3rd quadrant		
	21 brackets, 2 arch wires (*n* = 23)	With steel wire (*n* = 7)	With Ti–Mo wire (*n* = 7)	Without wire (*n* = 7)
*Dentists*				
Interrater reliability	0.992	0.987	0.990	0.997
Intrarater reliability	0.994–0.964 best/worst rater	0.983 summarized	0.954 summarized	0.986 summarized
*MTRA*				
Interrater reliability	0.979	0.984	0.956	0.995
Intrarater reliability	0.981–0.918 best/worst rater	0.939 summarized	0.945 summarized	0.976 summarized

*MTRA* medical-technical radiology assistants

The intrarater reliability was excellent for all study participants and over the 23 measurement points: The dentists tended to be slightly better than the MTRAs with α between 0.994 and 0.964 and between 0.981 and 0.918 respectively.

The calculation of validity (agreement between intraoral measured values and laboratory values) showed very strong positive correlations for all 23 measuring points for both dentists (Pearson correlation r = 0.967) and MTRAs (r = 0.922) (Table 5). Evaluation of these correlations [16] showed that the dentists had a better agreement with the laboratory values than the MTRAs (*p* = 0.002).

**Table 5:** Agreement of the clinically and in vitro determined permeability with joint and separate calculation of the quadrants with and without arch wires Übereinstimmung der klinisch und in vitro ermittelten Permeabilität mit gemeinsamer und separater Berechnung der Quadranten mit und ohne Drahtbögen

Pearson correlation	All measuring points (*n* = 23)	3rd quadrant without wires (*n* = 7)	1st and 2nd quadrant with metal wires (*n* = 14)
Dentists	0.967*	0.984	0.966**
MTRA	0.922*	0.960	0.917**

*MTRA* medical-technical radiology assistants

* *p* = 0.002, ** *p* = 0.009

Differences between the groups were found for the deviations of the mean values of the measurements from the laboratory values. Among the dentists, a very small, nonsignificant deviation of −0.003 was found. For the MTRAs, the deviation was −0.016. It differed both from the laboratory value (*p* = 0.011; T = −2.60; df = 91) and from the dentists’ results (*p* = 0.032; T = 2.18; df = 91).

To determine the influence of the two ligated arch wires, Cronbach’s α and the Pearson correlation were calculated separately for the quadrants with and without arcs (Tables 4 and 5).

**Fig. 5:**
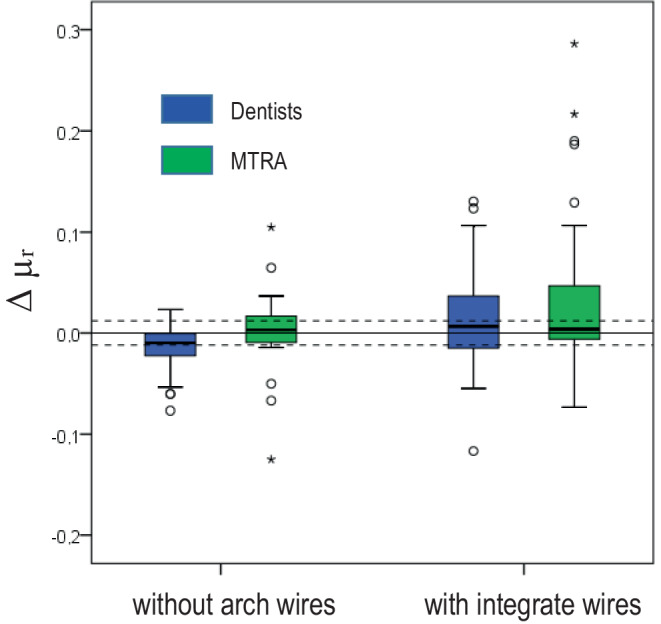
Average deviations (Δµ_r_) of the clinical permeability measurements from the laboratory values on brackets with and without arch wires. The *dashed* *line* shows the measurement deviation of ±0.012 Die gemittelten Abweichungen (Δµ_r_) der klinischen Permeabilitätsmessungen auf Brackets mit und ohne Drahtbogen von den entsprechenden Laborwerten. Die *gestrichelte* Linie zeigt die Messabweichung des Gerätes von ±0,012

The separate determination of the validity showed that the difference between dentists and MTRAs in the overall calculation was caused by the ligated arches, because this difference was not found in the individual examination of the 3rd quadrant (without arch wires). Overall, however, the agreement values and correlations consistently remained in the excellent or strongly positive range. Fig. 5 shows the scattering caused by the arch wires.

## Discussion

Until now, radiologists and orthodontists did not have a valid and on-site available database for deciding whether intraoral metal objects can remain in situ prior to a head and neck MRI. We proposed a direct measurement of the relative magnetic permeability with an appropriate device (Ferromaster) and investigated its applicability under intraoral conditions. With its upper measuring range limit of 1.999 it is suitable for all potential metals. They can be divided into two groups based on their magnetizability:

- Alloys based on titanium, cobalt and nickel as well as stable austenitic stainless steels with µ_r_ < 1.01
- Metastable austenitic or martensitic steels with µ_r_ up to 1.8.

Keepers for monomagnetic attachments are an exception as they are specifically manufactured from highly ferromagnetic alloys such as PdCo (µ_r_ ≈ 240). In these, the maximum value of the measuring range of 1.999 is displayed. We tested the accuracy of the measurement under the manufacturer’s application restrictions for undersized, nonplanar and remanently magnetizable objects. Due to its conformity to EN 60404-15 and ASTM 342, a high precision of the measuring device was to be expected. However, we were able to show that the precision is excellent even on clinically used small, nonplanar objects.

The trueness as a second accuracy criterion was decreased on our undersized objects. Nevertheless, we were able to show that the true (target) µ_r_ values of strongly undersized objects can be calculated with sufficient accuracy from the nonexact (actual) values by experimentally created correction formulas. The exemplary determined correction factors apply to the respective volume. However, the determined curves demonstrate that this is a proportionally systematic deviation which only occurs when leaving the starting point of the curves. This results in the following statement which is essential for a screening: independent of the object size and taking into account the measuring deviation, a measurement result of the Ferromaster of µ_r_ ≤ 1.002 is always correct.

The third manufacturer’s restriction to only use the measuring device on remanent objects after they have been demagnetized is intended to prevent the permanent magnet in the probe from being altered by external fields and thus rendering the device unusable. In fact, products 9–14 (Table 2) were already magnetized by exposure to the weak field of the probe, but these remanent fields did not further amplify after exposure to the main field of the MRI, which is about 40 times stronger. The first exposure of these soft magnetic steel types which are typically used for medical purposes already produced saturation magnetization, which can no longer be increased. The remanence of medical steels that occurs at magnetic saturation is far too low to affect the measuring probe. Thus, our study shows the irrelevance of this use restriction of the manufacturer for planned clinical application. The only exception are intraoral primary magnets of the duo-magnet attachments which are used for anchoring implant-supported dentures or epitheses. Their field can be up to 140 mT and would render the small magnets of the Ferromaster probe useless. Thus, anamnesis prior to MRI should involve asking for intraoral fixed mini magnets when the patient is wearing a removable prosthesis.

This is because MR exposure of such magnets leads to maximal artifacts and may further, depending on their position to B_0_, cause insufficiency of the attachments. When in doubt, a simple field sensor in the form of a small bar magnet, freely rotatable in all axes, can be applied to determine whether the object is a magnet or just a ferromagnetic product.

In the present study, we were able to show that possible artifacts can be better predicted with a direct permeability measurement than by knowing the present alloy. This is demonstrated by some of the products listed in Table 2: product No. 8 consists of two steels (DIN 1.4404 and 1.4301) with a metastable austenitic structure. Accordingly, µ_r_ was 1.00. The magnetizability declared in the steel standard and the measured magnetizability agree. This is due to the production process “metal injection moulding” (MIM), where the original microstructure is retained in the final product. Products No. 9, 10 and 11 are made of the same steel grades, but our measurements showed µ_r_ values up to 1.48. This massive discrepancy is due to local martensite formation caused by cold deformation or hardening during the production process, which makes them magnetizable [7, 15].

**Fig. 6:**
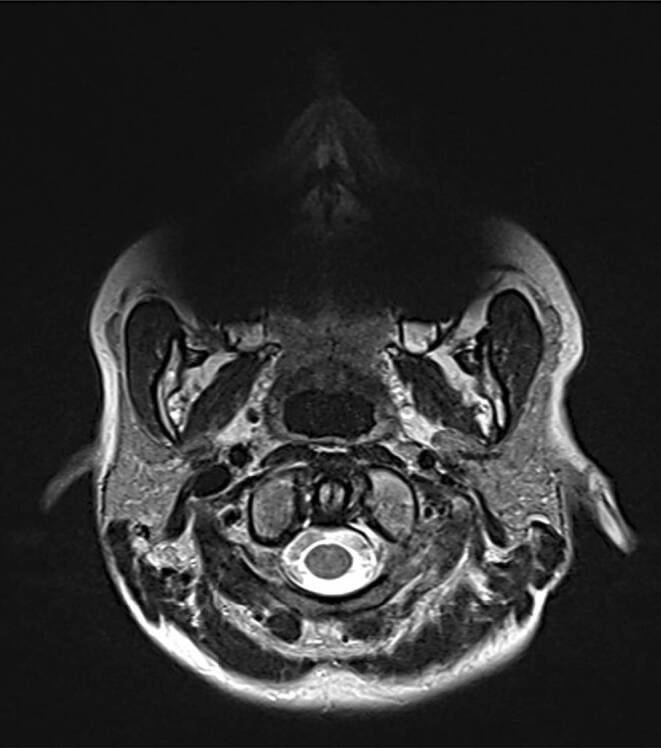
Patient A: MRI image (1.5 T): transversal section at the level of the *dens axis*, T2-weighted TSE sequence (TR: 5.700/TE: 112). Ceramic brackets on the anterior teeth, metal brackets on premolars and molars, made of the metastable austenitic steel grade 1.4301 (Dental-Vertrieb 2000 GmbH, Alsbach-Hähnlein, Germany). Measured permeability µ_r_ = 1.472. Limited assessability due to susceptibility artifacts Patient A: MRT-Bild (1,5 T): transversaler Schnitt auf Höhe des *Dens axis*, T2-gewichtete TSE-Sequenz (TR: 5.700/TE: 112). Keramikbrackets an den Frontzähnen, die Metallbrackets an den Prämolaren und Molaren sind aus dem metastabile austenitischen Stahl 1.4301 (Dental-Vertrieb 2000 GmbH, Alsbach-Hähnlein/Deutschland) hergestellt. Gemessene Permeabilität µ_r_ = 1,472. Eingeschränkte Beurteilbarkeit aufgrund von Suszeptibilitätsartefakten

**Fig. 7:**
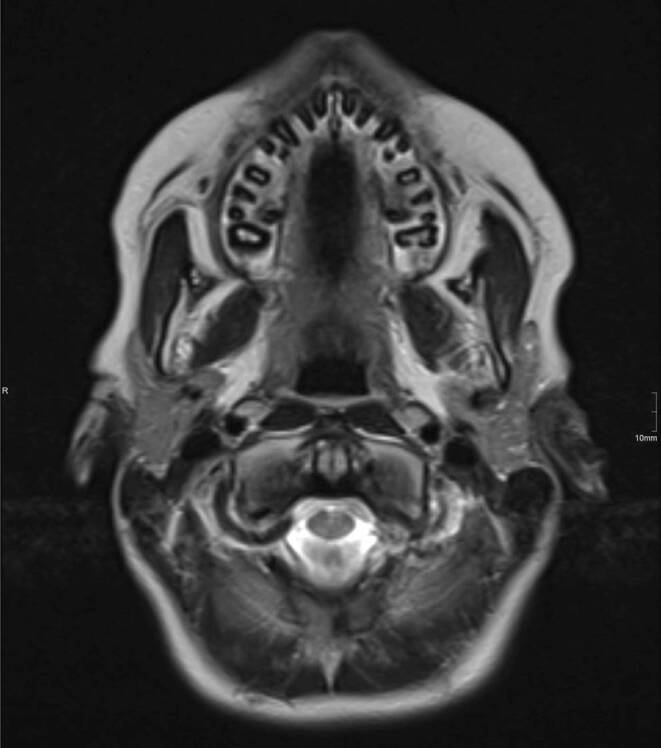
Patient B: MRI image (1.5 T): transversal section at the level of the *dens axis*, T2-weighted TSE sequence (TR: 5.400, TE: 125). Ceramic brackets on the anterior teeth and premolars, metal brackets on the molars, made of the metastable austenitic steel grades 1.4301, 1.4404 and 1.4501 (Dentaurum GmbH & Co. KG). Measured permeability µ_r_ = 1.001. Artifact-free imaging. The alveoli of the maxillary teeth fitted with brackets are also displayed without interference. (Image source: Dr. Gabriele Hahn, Dresden University Hospital) Patient B: MRT-Bild (1,5 T): transversaler Schnitt auf Höhe des *Dens axis*, T2 gewichtete TSE-Sequenz (TR: 5.400, TE: 125). Keramikbrackets an den Frontzähnen und Prämolaren, Metallbrackets an den Molaren, hergestellt aus den metastabilen austenitischen Stahlsorten 1.4301, 1.4404 und 1.4501 (Dentaurum GmbH & Co. KG). Gemessene Permeabilität µ_r_ = 1,001. Artefaktfreie Bildgebung. Auch die Alveolen der mit Brackets versehenen Oberkieferzähne sind störungsfrei dargestellt. (Bildquelle: Dr. Gabriele Hahn, Universitätsklinikum Dresden)

As an example, this is shown in two patients wearing similar orthodontic appliances in the posterior region during MR examination (Figs. 6 and 7): The metal brackets were made of metastable austenites of the same grade; however, the way the brackets were manufactured was very different. In patient A (Fig. 6), brackets were cold-formed products that triggered major artifacts. In patient B (Fig. 7), brackets had been manufactured using the MIM process and were not visible in the imaging.

On the other hand, a µ_r_ reduction is also possible without changing the official specification of a steel. For example, µ_r_ of grade 1.4301 can be reduced from 1.075 to 1.011 by solution annealing [10]. Products with this reduced value are considered nonferromagnetic and would not produce any relevant artifacts in the MRI. Since this state is not subject to declaration, it can only be found by direct measurement.

In a survey among radiologists in Germany, 40% of the participants were open to the idea of a clinical intraoral permeability measurement [14]. In the present study we have shown for the first time that this is feasible with excellent reliability and validity and without professional dental experience. The slightly poorer MTRA results could be further improved with feedback training (comparison with laboratory values). Because of the blinding effect, this was omitted in this study. The outliers observed in Fig. 5 were most likely due to positioning errors during the measurement on the ligated arch wires.

In order to prevent an unintended learning effect among the study participants and to exploit the full measuring range of the Ferromaster, we placed different brackets and tubes made of different alloys and from different manufacturers on the appliance of the test subject. In clinical reality, measurement should be even simpler, as the compilation of “real” orthodontic appliances is less diverse than the one used in this study.

The presented method can be used for all directly accessible and not instantly removable metal objects: For example, on activation rods of maxillofacial surgical distractors, on splints and bimaxillary fixations and, if applicable, on fixed intraoral or extraoral piercings.

The measurement meets the criteria for a physical screening, which means that it can filter out from a set of elements those that exhibit the required property. The detection of a µ_r_ value ≤ 1.002 reliably indicates the absence of magnetizability, regardless of the object size. We showed that within this range, the device was able to measure with the same high precision and trueness even on very small objects and without a correction factor. Furthermore, the quality criteria of medical screening tests can be applied, since sensitivity and specificity were 100% in this value range.

As µ_r_ values increase and depending on the distance between the objects and the region of interest, radiologists must then decide whether removal of the material is essential or whether less artifact-prone sequences or sequence modifications can allow the objects to remain intraorally. However, relevant artifact-reducing modifications are not yet available for all diagnostically required sequences.

Thus, future diagnostic studies should investigate the correlation between µ_r_ values ≥ to 1.002 and the artifact size that occur for a wide range of MR sequences.

## Conclusions

Even the few available manufacturer’s specifications on the magnetizability of dental and oral surgical metal products often convey a false impression. This property, however, is the crucial material-related predictor for unwanted interactions in MRI. The method for permeability measurement tested in this study using a commercial device slightly modified for this purpose can be utilized as a chair-side screening for artifact formation. Nonmagnetizable objects can be reliably distinguished from magnetizable objects. The precision of the measurement is high on all potential objects, and trueness is high for objects with µ_r_ ≤ 1.002. For objects with higher µ_r_ values, correction factors can be calculated.

Remanence induced by exposure to strong external magnetic fields does not affect these measurements. After simple instruction dentists and medical staff without clinical-intraoral experience can clinically apply it with excellent reliability and validity under “waiting room conditions”. The additional use of orthodontic arch wires increases the dispersion of the measured values while presenting the same high validity.

This method can replace the frustrating material research of dental products. It finally offers the radiologist a valid and rapidly available decision-making aid as to whether fixed metallic materials can remain completely in situ prior to MRI. This could avoid unnecessary material removal and concomitant expense and discomfort.

Also, contrary to the opinion expressed in many publications, a selective evaluation of individual metallic components is possible for the first time, since a reliable distinction can be made between nonpermeable and highly permeable parts.

A Germany-wide survey has shown that radiologists consult the treating orthodontist when in doubt about intraoral appliances [14]. We hope that this study can also promote interdisciplinary understanding between the two specialties. This is also important considering that MRI may become a diagnostic tool for orthodontics in the future, for example for cephalometric analysis [12].
